# Hernie inguinale de la vessie: à propos de 8 cas

**DOI:** 10.11604/pamj.2015.22.7.7474

**Published:** 2015-09-04

**Authors:** Alioune Sarr, Cyrille Ze Ondo, Yaya Sow, Boubacar Fall, Amath Thiam, Babacar sine, Rodrigue Djoufang, Babacar Diao, Papa Ameth Fall, Alain Khassim Ndoye, Mamadou Ba

**Affiliations:** 1 Service d'Urologie-Andrologie, Hopital Aristide Le Dantec, avenue Pasteur, Dakar, Sénégal

**Keywords:** Vessie, hernie, découverte peroperatoire, bladder, hernia, intraoperative finding

## Abstract

Décrire notre expérience de la prise en charge des hernies inguinales de la vessie (HIV). Il s'agit d'une étude rétrospective descriptive colligeant les dossiers des patients opérés pour une HIV entre janvier 2005 et décembre 2012. Les paramètres suivants ont été étudiés: l’âge des patients, les aspects anatomo-cliniques de la hernie, les circonstances de découverte, l'attitude thérapeutique et les résultats de la cure. Huit HIV ont été diagnostiquées sur une période de 7 ans. Tous les patients étaient de sexe masculin. La moyenne d’âge était de 57,6 ans. La HIV siégeait à droite chez 5 patients et était associée à une HBP chez 3 patients, deux patients avaient des antécédents de herniorraphie. La découverte était per opératoire chez 6 patients, postopératoire (fistule vesicocutanée) chez un patient et préopératoire chez un patient. Ce dernier a présenté une HIV géante diagnostiquée à l'Uroscanner. L'attitude thérapeutique était fonction des circonstances de découverte de la HIV et de la pathologie associée. Six patients ont été opérés selon la technique de Bassini et deux selon la technique de Mac Way. La durée moyenne de l'hospitalisation était de 7 jours. Après un suivi régulier de 2 ans nous n'avons pas noté de récidive herniaire. La HIV est une affection rare dont la découverte est le plus souvent per-opératoire après une taille vésicale. Il faut l’évoquer chez tout patient aux antécédents d'herniorraphie et chez les sujets âgés de plus de 50 ans qui présentent une hernie inguinale associée à des TUBA.

## Introduction

La hernie inguinale est l'une des pathologies les plus fréquentes en chirurgie et se définit par le passage du contenu abdominal ou pelvien à travers l'orifice inguinal [1]. On parle de hernie inguinale de la vessie (HIV) lorsque le contenu intéresse la vessie [2]. La HIV est une pathologie rare découverte le plus souvent en peropératoire [3]. A travers une série de 8 cas et une revue de la littérature nous allons discuter des aspects épidémiologiques, étiologiques, anatomo-cliniques, et thérapeutiques des HIV.

## Méthodes

Il s'agit d'une étude rétrospective, descriptive colligeant les dossiers des patients opérés pour une HIV au niveau du service d'urologie de l'hôpital Aristide Le Dantec entre janvier 2005 et décembre 2012. Les paramètres suivants ont été étudiés: l’âge des patients, les aspects anatomo-cliniques de la hernie, les circonstances de découverte, l'attitude thérapeutique et les résultats de la cure.

## Résultats

Le Tableau 1 résume nos différentes observations. Huit HIV ont été diagnostiquées sur une période de 7 ans. Tous les patients étaient de sexe masculin. La moyenne d’âge était de 57,6 ans avec des extrêmes de 27 ans et 70 ans. Six patients étaient âgés de plus de 50 ans. La HIV siégeait à droite chez 5 patients. Deux patients avaient des antécédents de herniorraphie.

**Tableau 1:** Récapitulation de nos différentes observations

N°	Cote	Age(ans)	MC	ANT	P.A	CDD	Traitement
1	D	60	Issue d'urine		-	FVC	Drainage vesical
2	G	65	Tumefaction inguinoscrotale	H	-	TDM	RV+ Bassini
3	D	70	TUBA + tumefaction inguinale		HBP*( VP= 60cc, TPSA = 1,2 ng/ml)	DPO	Cystorraphie + RV + Bassini + Drainage
4	G	68	TUBA + tumefaction inguinale		HBP**( VP= 67cc, TPSA = 2,5ng/ml)	DPO	RV + Bassini + ADENOmec
5	D	62	TUBA + tumefaction inguinale		HBP**( VP= 80cc, PSA = 3,8 ng/ml)	DPO	RV + Bassini + ADENOmec
6	G	40	Tumefaction inguinale		-	DPO	Cystorraphie + RV + Mac Way +Drainage
7	D	38	Tumefaction inguinoscrotale	H	-	DPO	Cystorraphie + RV + Bassini + Drainage
8	D	58	Tumefaction inguinale		-	DPO	Cystorraphie + RV + Bassini + Drainage

**G**:gauche **TUBA**: trouble urinaire du bas appareil **FVC**: fistule vesicocutanée

**HBP**: hypertrophie bénigne de la prostate **VP**: volume prostatique **H**: Herniorraphie **TDM**: tomodensitometrie **DPO**: découverte peroperatoire **RV**: refoulement de la vessie **ADNOmec**: adenomectomie **CC**: centimètre cube

* Bonne réponse aux alpha bloquants

** Absence de reponse aux alpha-bloquant

Les motifs de consultations étaient une tuméfaction inguinale ou inguino-scrotale isolée chez 4 patients et une tuméfaction inguinale associée à des troubles urinaires du bas appareil (TUBA) chez trois patients. Un patient opéré pour hernie inguinale gauche était revenu consulter pour une issue d'urines par la plaie opératoire. Chez les trois patients qui étaient venus consulter pour une tuméfaction inguinale associée à des TUBA, l'examen clinique avait permis d'objectiver une prostate augmentée de volume d'allure bénigne associée à une hernie inguinale non compliquée. Le taux de PSA était inferieur à 4 ng/ml chez ces patients et le volume prostatique moyen était à 69 cm^3^. Un traitement à base d'alpha-bloquants a été mis en route chez ces patients. Devant l’échec du traitement médical, chez deux d'entre eux, l'indication d'une adénoméctomie prostatique associée à une herniorraphie a été posée par contre chez le troisième patient qui a bien répondu aux alpha-bloquants, nous avons procédé uniquement à une herniorraphie.

**Figure 1:**
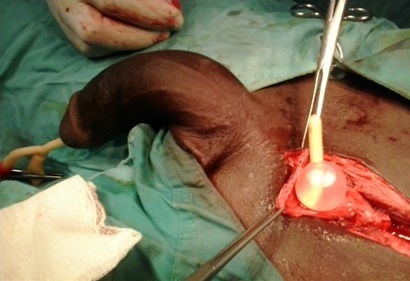
Ouverture accidentelle de la vessie (confondue à un sac herniaire péritonéal)

La découverte d'une HIV était per opératoire chez 6 patients, devant l'ouverture accidentelle de la vessie (confondue à un sac herniaire péritonéal) (Figure 1).

**Figure 2:**
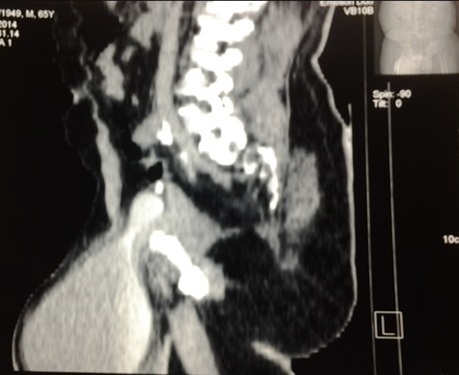
Coupe sagittale d'une UroTDM révélant une cystocéle inguino-scrotale

Le diagnostic était posé en pré-operatoire chez un patient. Il s'agissait d'un patient aux antécédents de herniorraphie droite qui était venu consulter pour une tuméfaction inguino-scrotale droite. L'examen physique avait permis de mettre en évidence une volumineuse hernie inguino-scrotale indolore dont la pression déclenchait l'envie d'uriner. La réalisation d'une tomodensitométrie pelvienne avait permis de mette en évidence une cystocéle inguino-scrotale (Figure 2). Le diagnostic était post-operatoire chez un patient qui avait présenté dans les suites immédiates d'une cure de hernie inguinale selon la technique de Mac Way, une issue d'urines à travers la kelotomie.

Chez les 6 patients qui ont eu une ouverture accidentelle de la vessie, nous avons réalisé une cystorraphie par un surjet aller-retour au fil résorbable 0 suivie d'un refoulement de la vessie dans l'espace de Retzius associée à un drainage transuretral de la vessie pendant une durée moyenne de 7 jours. Nous avons procédé à un refoulement de la vessie associé à une herniorraphie chez le patient qui avait une découverte pré-operatoire de la hernie. Le patient qui a présenté une fistule vésico-cutanée après cure d'une hernie a eu un drainage vésical pendant 7 jours ce qui a permis un tarissement de la fistule. il s'agissait d'une hernie oblique interne chez tous les patients. La réfection pariétale était réalisée selon la technique de Bassini chez 6 patients et selon Mac Way chez 2 patients. La durée moyenne de l'hospitalisation était de 7 jours. Après un suivi régulier de 2 ans, nous n'avons pas noté de récidive herniaire.

## Discussion

La HIV est une entité rare puis qu'elle ne représente que 1 à 4% des hernies inguinales [4]. Elle résulte de l'association d'une faiblesse pariétale à une élévation de la pression intra-abdominale. Les facteurs prédisposant à une HIV sont l'obésité, une faible musculature de la paroi abdominale et surtout un obstacle sous vésical [5]. Plus qu'une sténose de l'urètre, l'obstacle sous vésical est le plus souvent une hypertrophie de la prostatique ce qui explique la fréquence de la HIV dans la tranche d’âge 50 ans-70 ans [6]. Dans notre série, six patients sur huit avaient plus de 50 ans et l'HBP était associée à la HIV chez trois patients. La HIV a été également rapportée après une chirurgie en particulier une herniorraphie et Server et al incriminent une traction des sutures sur la paroi vésicale ou le péritoine dans la genèse des HIV [7]. Ceci pourrait expliquer la survenue de HIV chez deux de nos patients, aux antécédents de herniorraphie qui ne présentaient pas apparemment un obstacle sous vésical.

Bien que plus fréquente chez l'homme, du fait de la fréquence de la pathologie prostatique, 30% des HIV se développent chez la femme [6]. Comme dans notre série, la HIV réalise habituellement une hernie oblique interne qui siège à droite et n'intéresse qu'une partie de la vessie (corne ou diverticule) [3, 8, 9]. La hernie inguinale géante de la vessie décrite chez notre deuxième patient a été rarement rapportée dans la littérature [5, 10] La HIV est le plus souvent asymptomatique. Parfois, elle s'accompagne de TUBA qui ne sont pas spécifiques et sont le plus souvent en rapport avec une hypertrophie prostatique associée [2, 3]. Le classique signe de Mery qui se traduit par une miction en deux temps, facilitée par l'appui sur la voussure herniaire et la disparition de la hernie après la vidange vésicale, constitue un signe clinique très évocateur mais inconstant [4]. L'imagerie médicale peut aider à poser le diagnostic de HIV en préopératoire Le plus souvent il s'agit d'une UCR réalisée à la recherche d'une sténose de l'urètre qui objective la HIV, sous la forme d'une image d'addition au contour vésical, unilatérale, régulière, arrondie, communiquant largement avec la vessie et descendant sous le plancher vésical [2]. Outre l'UCR, l’échographie ou mieux l'Uro-TDM peut poser le diagnotic de HIV [11]. Mais en pratique quotidienne, ces examens d'imagerie ne sont pas demandés, l'attitude la plus courante étant d'opérer directement toute hernie diagnostiquée [1]. Ainsi la HIV est découverte en préopératoire que dans 7% des cas [6] tandis que dans 16% des cas, elle est diagnostiquée en postopératoire devant une suppuration pariétale ou une fistule vésico-cutanée [6] comme c'est le cas chez notre premier patient. La fistule vesicocutanée est due à une brèche vésicale méconnue tandis que la suppuration pariétale est secondaire à la diffusion d'urines infectées, transformant ainsi une chirurgie propre en une chirurgie propre-contaminée.

Classiquement, la HIV est découverte après une taille accidentelle de la vessie. Cette découverte fortuite peut être lourde de conséquences puis qu'elle entraîne un allongement du séjour hospitalier, sans compter que le drainage vésical transuretral peut se compliquer d'une sténose iatrogène de l'urètre. Dans notre courte série, la HIV était découverte en peropératoire dans 75% des cas. Nos résultats sont proches de ceux de Watson et al qui avaient noté 80,4% de découverte peropératoire sur une sérié de 347 HIV [6]. Comme toute hernie, la HIV peut s’étrangler ceci d'autant plus que le collet est étroit, mais l'essentiel de ces complications sont en rapport avec la stase urinaire (infection urinaire, lithiase vésicale) [12] des complications plus rares ont été rapportées telles que la survenue d'une tumeur vésicale intraherniaire [13] ou une insuffisance rénale secondaire à la compression du trigone [5]

En cas de diagnostic pré-opératoire le traitement d'une HIV ne diffère pas de celui des autres hernies et consiste à un refoulement de la vessie associée à une réfection de la paroi. La résection de la vessie est à proscrire du fait du risque de réduction de la capacité vésicale et de lésion urétérale [14]. Cependant la résection vésicale est indiquée en cas de volumineuse hernie, de collet étroit, de nécrose [8] et de tumeur intra herniaire [13]. Du fait de la dysurie qui entraîne une hyperpression abdominale, l'HBP est un facteur herniogène. La prévalence de la hernie inguinale est de 15% à 25% chez les patients admis pour cure d'adénome de prostate [15]. Devant cette association, il faut traiter l'HBP, en première intention, par des alpha-bloquants. En cas d'amélioration satisfaisante de la miction, comme chez notre troisième patient, il convient de réaliser une herniorraphie [3].

Par contre en cas d’échec du traitement médical, la chirurgie s'impose. Il peut s'agir d'une chirurgie ouverte (adénoméctomie prostatique transvésicale ou retro pubienne) ou endoscopique (résection transuretrale de la prostate) associée à une herniorraphie. La chronologie de ces deux actes est sujette à controverse, dans notre pratique quotidienne, nos procédons dans le même temps opératoire à une herniorraphie suivie d'une adénoméctomie prostatique, le plus souvent transvésicale [16]. Cette prise en charge simultanée à de nombreux avantages tels que la réduction du nombre d'interventions et d'anesthésie chez un sujet âgé, la réduction du coût économique et un meilleur confort psychologique [17].

## Conclusion

La HIV est une affection rare dont la découverte est le plus souvent per-operatoire après une taille vésicale. Il faut l’évoquer chez tout patient aux antécédents d'herniorraphie et chez les sujets âgés de plus de 50 ans qui présentent une hernie inguinale associée a des TUBA.
